# Mannose-Binding Lectin Gene Polymorphism and Its Association with Susceptibility to Recurrent Vulvovaginal Candidiasis

**DOI:** 10.1155/2018/7648152

**Published:** 2018-04-04

**Authors:** Noha M. Hammad, Nissreen E. El Badawy, Ashraf M. Nasr, Hamed A. Ghramh, Laila M. Al Kady

**Affiliations:** ^1^Department of Medical Microbiology and Immunology, Faculty of Medicine, Zagazig University, Zagazig, Egypt; ^2^Department of Obstetrics and Gynecology, Faculty of Medicine, Zagazig University, Zagazig, Egypt; ^3^Research Center for Advanced Materials Science (RCAMS), King Khalid University, P.O. Box 9004, Abha 61413, Saudi Arabia; ^4^Department of Biology, Faculty of Science, King Khalid University, P.O. Box 9004, Abha 61413, Saudi Arabia

## Abstract

Recurrent vulvovaginal candidiasis (RVVC) is a common illness influencing childbearing women worldwide. Most women suffering from RVVC develop infection without specified risk factors. Mannose-binding lectin (MBL) is an important component of innate immune defense against* Candida *infection. Innate immunity gene mutations and polymorphisms have been suggested to play a role in susceptibility to RVVC. This study aimed to investigate the association between* MBL 2 *gene exon 1 codon 54 polymorphism and susceptibility to RVVC in childbearing women. Whole blood and serum samples were obtained from 59 RVVC cases and 59 controls. MBL serum level was measured by enzyme-linked immune-sorbent assay (ELISA).* MBL2 *exon 1 codon 54 polymorphism was determined by polymerase chain reaction-restriction fragment length polymorphism (PCR-RFLP). It was shown that MBL serum level was nonsignificantly different between RVVC cases and controls. The risk of RVVC was 3 times higher in those carrying* MBL2 *exon 1 codon 54 variant allele (B). It could be concluded that the carrying of* MBL2 *exon 1 codon 54 variant allele (B) was shown to be a risk factor for RVVC in childbearing women.

## 1. Introduction

Vulvovaginal candidiasis (VVC) is the second most common vaginal infection after bacterial vaginosis [1]. It is diagnosed in up to 40% of women with vaginal complaints in the primary care setting [2–4]. Nevertheless, it was estimated that 3 out of 4 women were likely to endure at least one episode of VVC in their lifetime. Moreover, about 5−10% of those women were suffering from recurrent infections [5]. Recurrent VVC (RVVC) is a multifactorial illness with possible underlying risk factors such as diabetes mellitus, antibiotic use, or pregnancy, but most of women with RVVC develop infection without identifiable risk factors [4]. RVVC can be defined as four episodes or at least three episodes unrelated to antibiotic therapy and occurring within one year [6]. The question about what factors determine which women experience transition from infrequent VVC to RVVC has not been resolved yet [7, 8]. Therefore, different mutations and polymorphisms in innate immune genes can alter the vaginal mucosal defense mechanisms against* Candida *species [9].

Mannose-binding lectin (MBL) is an important component of the innate immune system. MBL is originally synthesized in the liver, circulates throughout the body, and has the ability to identify a broad range of pathogens [10]. MBL is capable of binding to mannan fraction of* Candida *cell wall, activating complement pathway, and hence, might reduce systemic infections by* Candida* spp. and in turn vaginal colonization [11]. Furthermore, macrophages and dendritic cells express receptors that can recognize MBL, facilitating opsonization of microorganisms with bound MBL on their surface [12, 13].

Mannose-binding lectin is a large macromolecule that has a bouquet-like structure. The basic structural subunit of MBL is a homotrimer of MBL polypeptides, entwined in a triple helix. Each single polypeptide chain has four domains: (1) a 21-amino acid N-terminal cysteine-rich region involved in oligomerization, (2) a 59-amino acid collagen-like domain, (3) a 30-amino acid *α*-helical, hydrophobic coiled-coil neck domain, which is crucial for initiating the oligomerization, and (4) a 188-amino acid C-terminal carbohydrate recognition domain [14, 15].

Three common structural polymorphisms caused by single point mutations are found in* MBL2 *gene and all are present on exon 1: allele “D” at codon 52 which is C to T nucleotide substitution, allele “B” at codon 54, and allele “C” at codon 57, and both are G to A nucleotide substitutions. All three mutations occur within the collagen domain changing the ability of MBL to oligomerize. The wild type is termed allele “A” while the structural variants B, C, and D are often gathered and referred to as zero “0” [16–18]. It has been proposed that polymorphisms in* MBL2 *gene are involved in determining susceptibility to RVVC [6]. However, the role of* MBL* genes polymorphism remains controversial and has not been determined by all investigators yet [19]. A similar work was carried out [6, 16–19]. However, none of them discussed Egyptian patients. To fill this gap, this study would give the chance to investigate* MBL* codon 54 polymorphism among childbearing Egyptian women complaining of RVVC.

The aim of this study was to investigate the potential role of MBL serum level and* MBL*2 gene exon 1 codon 54 polymorphism in determining susceptibility to RVVC in childbearing Egyptian women. Therefore, it could be possible to explore new potential therapeutic modalities for MBL-deficient women suffering from RVVC.

## 2. Materials and Methods

This study was conducted at Immunology Research and Molecular Biology Laboratories, Microbiology and Immunology Department, Faculty of Medicine, Zagazig University, Zagazig, which is located 100 kilometers northeast of Cairo, the capital of Egypt. This study was carried out in the period of December 2014 to May 2017.

### 2.1. Ethical Approval

This study was approved by the institutional review board, Faculty of Medicine, Zagazig University, Zagazig, Egypt. The study subjects were informed, preliminary, about the nature and the purpose of the study. The written informed consent was taken from all participants. The study subjects were not exposed to any harm or risk. Participants' data were confidential.

### 2.2. Subjects

The present study enrolled 59 childbearing women (age, 30–40 years). Three replicates of vaginal swabs were taken from women that were suffering from suspected VVC and were exposed to at least three episodes of VVC in the last 12 months. Control subjects were 59 healthy women (age, 30–40 years) without previous history of* Candida *infection or gynecologic complaints.

All RVVC cases and controls were seen at Zagazig University Hospitals and Qenayat Hospital Gynecology Outpatient Clinics, Qenayat, Egypt (neighbor to Zagazig City). RVVC cases and controls were matched for age, marital status, and socioeconomic status. The sociodemographic variables of the study subjects were collected by a questionnaire. The genital hygiene behavior of the study subjects was assessed by genital hygiene questionnaire. The exclusion criteria for this study were pregnancy, contraception, sexual activity and vaginal douching in the last week, steroid therapy for less than 3 months, diabetes, immunodeficiencies, and immunosuppressive therapy.

### 2.3. Vaginal Swabs and Identification of* Candida *Species

Vaginal swabs were obtained using sterile cotton-tipped plastic swabs and cultured onto Sabouraud Dextrose Agar Medium (Sabouraud Dextrose Agar, Oxoid, UK) and then subcultured on chromogenic agar medium (CHROMagar™ Candida, Paris, France) for presumptive identification of species. The vaginal samples were also tested for* Trichomonas vaginalis* by wet mount and for bacterial vaginosis by Amsel criteria [20]. Cultures were examined under light microscope to show the budding yeast cells with or without pseudohyphae, blastospores, and germ tubes [3, 21]. In addition, biochemical tests were studied using Hi-Candida™ API identification kit (Biomereux, France).

### 2.4. Blood Sampling

Three mL of peripheral blood was obtained from each study participant by venous puncture, collected and divided into 2 (13 × 75 mm) tubes, EDTA containing tube and Wassermann's tube, and stored at −20°C until used. Blood collected in EDTA tube was subjected to subsequent direct blood PCR. The blood collected in Wassermann's tube was centrifuged at 3000 RPM for 10 min and the supernatant serum was collected for subsequent determination of MBL serum level.

### 2.5. Quantitation of Serum MBL

MBL serum level was measured by sandwich enzyme-linked immunosorbent assay (ELISA) according to the manufacturer's company protocol (Quantikine® ELISA Human MBL; R&D Systems, Minneapolis, USA).

### 2.6. Determination of* MBL2 *Gene Polymorphism

*MBL2 *gene exon 1 was amplified by direct blood PCR (Phusion™ Blood Direct PCR Master Mix; Thermo Scientific™, USA). PCR reactions were performed in 50 *μ*L final volume using 0.5 *μ*M of each primer (forward: 5′-TAGGAC AGAGGGCATGCTC-3; reverse: 5′-CAGGCAGTT TCCTCTGGAAGG-3′ PCR product size 349 bp), 25 *μ*L of 2X Phusion Blood Direct PCR Master Mix, and 5 *μ*L whole blood. After an initial lysis of cells for 5 min at 98°C, PCR reactions were run for 40 cycles including 5 s at 98°C, 30 s at 58°C, and 30 s at 72°C with a final extension at 72°C for 1 min. PCR products were loaded directly into pits of 1.5% agarose gel and analyzed by electrophoresis. The obtained PCR products were digested with restriction enzyme, Ban I according to the manufacturer's company protocol (BshNI; Thermo Scientific, USA). The wild allele “A” was cut into two fragments, 260 and 89 bp, while the variant allele “B” remained uncut. The digested products were loaded on 2% agarose gel and analyzed by electrophoresis.

### 2.7. Statistical Analysis

Quantitative data were represented as mean value ± 1 standard deviation (SD), median and range. Genotype and allele frequencies were determined by direct counting. Associations between MBL genotype or alleles and clinical variables were analyzed by Pearson Chi-Square (*χ*^2^) and Fisher's exact tests. The strength of association of* MBL2* exon 1 codon 54 genotypes and frequency of RVVC was calculated by estimating odds ratios (OR) for matched data at confidence interval (CI) 95%. All tests were 2-tailed. Mann–Whitney *U* and Kruskal-Wallis tests were used for calculation of median difference between independent groups. Results were considered statistically significant when *P* (probability) values were equal to or less than 0.05. All analyses were performed using Statistical Package for the Social Sciences software version 24 (SPSS version 24, Inc., Chicago, IL, USA.).

## 3. Results

All RVVC cases and controls were negative for* Trichomonas vaginalis *and bacterial vaginosis; controls were also negative for* Candida *spp. All the vaginal swabs taken from the 59 RVVC childbearing women showed fungal growth on both Sabouraud Dextrose Agar and chromogenic agar. Further identification of* Candida *spp. by light microscopic examination and API identification kit revealed that 88.1% of the isolates were identified as* C. albicans *while 8.5% and 3.4% of the isolated were identified as* C. glabrata* and* C. tropicalis, *respectively.

**Figure 1:**
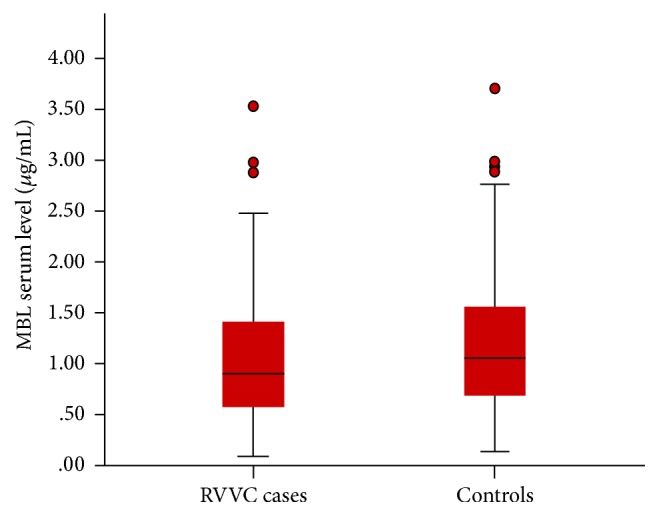
*Box plot showing the difference between RVVC cases *(*n* = 59)* and controls *(*n* = 59)* regarding MBL serum level.* The median MBL serum level of RVVC cases was nonsignificantly lower than that of controls (*P* = 0.145). The upper and lower ends of boxes and inner lines correspond to the upper and lower quartiles and median values, respectively. Whiskers indicate minimum and maximum values, and circles denote outliers.

No statistically significant difference in MBL serum level was observed between RVVC cases and controls (*P* = 0.145) (Figure 1). The median MBL serum level in RVVC cases was 0.90 *μ*g/mL (range, 0.09–3.53 *μ*g/mL) compared with 1.05 *μ*g/mL (range, 0.14−3.70 *μ*g/mL) in controls.

**Figure 2:**
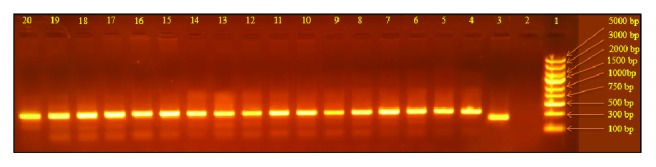
*PCR product of exon 1 of MBL2 gene amplification*. PCR products were electrophoresed on a 1.5% agarose gel and visualized under ultraviolet light by ethidium-bromide staining. Lane 1 is DNA Ladder, lane 2 is master mix as negative control, lane 3 is universal control of amplicon size 237 bp, lane 4 is positive control of purified human genomic DNA, lanes 5 to 12 are controls, and lanes 13 to 20 are RVVC cases. MBL gene amplicon size is of 349 bp.

As given in Figure 2, the molecular sizes (349 bp) of PCR products from RVVC cases (lanes 13 to 30) were parallel to those from the controls (lanes 5 to 12). This showed a successful process of PCR technique for amplification and detection of exon 1 of* MBL2 *gene.

**Figure 3:**
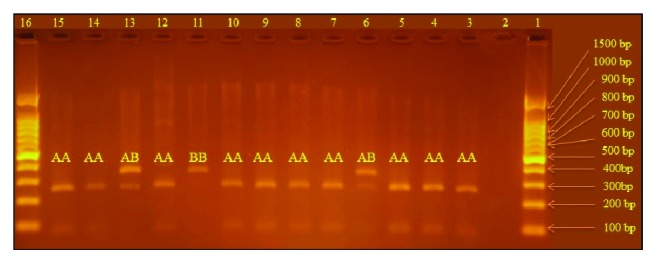
*MBL genotyping by RFLP.* Digested products were electrophoresed on 2% agarose gel and visualized under ultraviolet light by ethidium-bromide staining. Lanes 1 and 16 are DNA Ladder. Lane 2 is negative control. The wild* MBL *genotype (AA) is cut into two fragments with BanI enzyme, 89 and 260 bp, seen in lanes 3–5, 7–10, 12, 14, and 15. The mutant homozygous* MBL* genotype (BB) remains uncut, 349 bp, seen in lane 11. The heterozygous* MBL* genotype (AB) is seen in lanes 6 and 13.

PCR products digested by Ban I restriction enzyme produced two fragments of 260 and 89 bp for wild* MBL *genotypes (AA), three fragments of 349, 260, and 89 bp for heterozygous* MBL* genotypes (AB), and one uncut fragment of 349 bp for mutant homozygous* MBL* genotypes (BB) (Figure 3).

The distribution of MBL genotypes and alleles was significantly different between RVVC cases and controls (*P* = 0.038 and 0.013, resp.). Allele A (wild allele) was present, respectively, in 83.9% of RVVC cases and in 94.0% of controls, whereas allele B (mutant allele) was present in 16.1% of RVVC cases and in 6% of controls. No homozygous mutant genotype (BB) was found among controls. The risk of RVVC is 3.04 times higher among those who carried variant allele “B” in comparison to those who did not (Table 1).

Table 2 shows that, in the presence of wild* MBL *genotype, the risk of RVVC associated with bad genital hygiene behavior was 3.47 times higher than that associated with good genital hygiene behavior (*P* = 0.004) while in the presence of mutant* MBL* genotype the risk was increased to 18.67 times (*P* = 0.021).

In RVVC group, the mutant types (AB and BB) MBL serum levels were significantly lower than the wild type (AA) MBL serum level (*P* = 0.019 and 0.033, resp.). However, in control group, no statistically significant difference was found between mutant type and wild type MBL serum levels (*P* = 0.23) (Table 3).

## 4. Discussion

Vulvovaginal candidiasis is one of the most prevalent vaginal infections and represents approximately 40%–50% of all cases of infectious vulvovaginitis [22]. The prevalence of RVVC among childbearing women and its importance as an Egyptian public health problem make an interest to continue research on such cases to add deep knowledge on RVVC and to understand the behavior of its pathogen and its epidemiology within Egyptian patients [23]. RVVC and its control by MBL were studied previously, but unfortunately none of the published papers discussed such cases in Egyptian patients. In an attempt to fill this gap, this study was designed on 118 childbearing Egyptian women to find out new therapeutic strategy for RVVC.

The suspected* Candida *isolates were identified as described previously [3, 4, 24]. In the present study,* C. albicans *was the most prevalent (88.1%)* Candida *spp. isolated from RVVC cases, followed by* C. glabrata* (8.5%) and* C. tropicalis *(3.4%), respectively. This study agreed with most studies worldwide which have reported that* C. albicans *is the main infectious* Candida* spp. implicated in infections of RVVC (76 to 89%). The overall percentage of non*-albicans Candida *spp. ranges from 11% to 24% [25–28].

Genetic factors often play an important role in primary or idiopathic RVVC occurring in women without any recognizable risk factors. Moreover, the recurrence of acute attacks of VVC triggered by known risk factor is strongly relevant to genetic predisposition [29]. The understanding of such genetic factors that determine susceptibility to RVVC is crucial for future therapeutic modalities in these patients [30]. Variation of MBL concentration in cervicovaginal fluid depends on individual's MBL genotype [12]. The strong binding of* Candida* spp. to MBL suggested the importance of this protein in host defense against VVC [13].

The present study found no statistical significant difference in MBL serum level between RVVC cases and controls. This finding is supported by a previous study that found no significant difference when comparing MBL serum levels between RVVC cases and controls [31]. However, some investigators found that MBL serum level was higher in RVVC cases when compared to controls indicating that MBL might be defensive against RVVC [13]. On the contrary, other studies reported that MBL level was lower in RVVC cases compared to controls [12, 32]. Nevertheless, they depended on measurement of MBL in vaginal fluid while, in the present study, MBL was measured in serum. From practical point of view, determination of MBL level in vaginal discharge is technically too difficult to perform and to standardize. Even more, the entity of vaginal fluid is changeable throughout menstrual period [33].

The present study could find an association between* MBL2* exon 1 codon 54 polymorphism and susceptibility to RVVC. Carriage of mutant allele “B” was more frequent in women with RVVC (16.1%) than in controls (6%). In previous studies,* MBL2* codon 54 polymorphism has been associated with increased frequency of RVVC in Latvian, Brazilian, Chinese, and Belgium [12, 32, 34, 35] but not Italian patients [31].

In the present study, analysis of genital hygiene behaviors among wild MBL genotypes estimated that bad genital hygiene behaviors increased the risk of RVVC by 3.47 times while the risk of RVVC has been elevated to 18.67 times in the presence of mutant* MBL *genotypes. These results supported the fact that RVVC is monomicrobial; however, it is multifactorial disease in origin [36]. Moreover, good genital hygiene behaviors might be protective against RVVC in genetically predisposed women [37].

Furthermore, this study has reported that* MBL2 *codon 54 polymorphism was significantly associated with reduction in MBL serum levels among RVVC cases; however, such reduction was statistically nonsignificant among controls. This finding was in agreement with Milanese et al. [31] Consistently,* MBL2* codon 54 polymorphism has been associated with reduced vaginal concentrations of MBL [12, 38].* MBL2* codon 54 polymorphism results in either nonfunctional monomers in homozygotes or low functional serum levels of protein with shorter half-lives that are easily degraded to lower oligomeric forms in heterozygotes [39]. It has been estimated that healthy individuals who are homozygous for the wild type alleles (A/A) have MBL serum levels above 1 *μ*g/mL while heterozygous individuals (A/0) have serum levels ranging from 0.5 to 1 *μ*g/mL. However, homozygous individuals for the variant* MBL2* alleles (0/0) have MBL serum concentration below 0.05 *μ*g/mL [40]. Furthermore, MBL deficiency was defined before by plasmatic protein levels below 0.5 *μ*g/mL or by an MBL function lower than 0.2 U/*μ*L C4 deposition [41].

In addition, other structural and promoter* MBL*2 gene polymorphisms have been identified resulting in 7 common secretor haplotypes that eventually determine the serum MBL concentrations. HYPA, LYQA, and LYPA haplotypes are associated with high serum level and LXPA, HYPD, LYPB, and LYQC haplotypes are associated with low serum level. Therefore, it is conceivable that one patient with wild type allele for MBL codon 54 may have a promoter combination, for example, LXP haplotype that downregulates MBL production resulting in low MBL serum level [39]. However,* MBL2 *gene structural polymorphisms alone could be responsible for the defective binding of MBL to* Candida* via its lectin domain at early phase of vaginal infection by the fungus [42].

Interestingly, it was previously documented that both wild type and mutant type MBL vaginal levels were significantly higher in acute VVC than in controls but not in RVVC, suggesting that MBL may increase during first episode of VVC, albeit subsequent episodes may make the immune system less sensitive to* Candida* [32]. Moreover, there is an emerging hypothesis that both acute VVC and RVVC stem from different host reaction modulated by* Candida. *Acute VVC associated with high numbers of* Candida *results in immunosuppressive reaction of the host while RVVC being associated with low numbers of* Candida *generates hypersensitivity reaction in genetically predisposed women. The host reactions were linked to modulators resulting from interaction between mast cells and substances such as MBL involved in mycotic infections. That hypothesis introduced a concept of “vaginal cutoff” which is the minimum quantity level of the* Candida* above which it would activate an allergic reaction that justifies the recurrence of episodes of VVC, in vulnerable subjects [43].

Data from previous reports of Babula et al. hypothesized that women's genetic capacity to produce MBL and IL-4 influenced their susceptibility to RVVC. They stated strong inverse relationship between vaginal concentrations of IL-4 and anticandidal compounds, MBL and nitric oxide metabolites. Additionally, the homozygous variant allele IL-4^*∗*^T was associated with >2-fold increase of IL-4, >3-fold decrease of nitric oxide, and >2-fold decrease of MBL concentrations in vaginal fluid [12, 44].

Since MBL serum levels could be affected by infections, hormone and drug intake, several investigators chose MBL genotyping over MBL serum level. They observed that MBL serum level is strongly associated with* MBL2 *gene polymorphisms [45, 46]. Nevertheless, MBL serum levels are not affected by age, circadian rhythm, and physical exercise and, during inflammation, do not increase over 3-4-fold compared to baseline level unlike other acute phase reactants like C-reactive protein which increases sharply from 10-fold to 1000-fold [40, 47]. Despite the fact that MBL serum measurement is widely diffused as a diagnostic test, there are not standard guidelines able to determine which patient is needed to be tested [40, 48].

Taken together, these data suggest that other factors such as cytokine levels, other alleles, or additional polymorphisms are in tight linkage disequilibrium that may affect the level of MBL. Consequently, some observations in this study could be explained, such that some low serum MBL levels were associated with wild genotype (AA) as well as the nonsignificant difference in MBL serum level between RVVC cases and controls and between different genotypes among controls.

Collectively, MBL genotype of a person offers only a general idea of the expected plasma concentration and different combinations of haplotypes are associated with a wide range of MBL concentrations. Genotypes are fairly good indicators of the average MBL concentrations at population level albeit less reliable predictors for plasma MBL at individual level [49, 50].

As RVVC may not be substantially treated; therefore, further deciphering of vaginal host defense mechanisms against* Candida* becomes essential to design novel immunotherapeutic strategies to improve and/or substitute the usual antifungal treatments. Further work in this approach focusing on MBL protein and its direct impact on* Candida spp. *was conducted* in vitro* (results have not been published yet). The efficacy of MBL protein as a potential therapeutic agent against RVVC, particularly among MBL-deficient women, should be clearly investigated.

## 5. Conclusion

Women carrying the variant allele “B” of* MBL2* codon 54 polymorphism have more risk of developing RVVC. Therefore, MBL genotypic analysis can be used as surrogates for MBL serum levels in order to identify MBL-deficient women for alternative therapeutic options.

## Figures and Tables

**Table 1:** MBL genotypes and allelic frequency distribution among RVVC cases and controls.

Variable	RVVC cases *n* = (59)		Controls *n* = (59)		Test of significance	*P* value	OR (95% CI)
	*n*	%	*n*	%			
Genotypes							
AA	42	71.2	52	88.1	*Fisher's exact*	**0.038** ^*∗*^	**2.65** ^*∗∗*^
AB	15	25.4	7	11.9			
BB	2	3.4	0	0.0			

Alleles							
A	99	83.9	111	94.0	**χ** ^2^ **3.75**	**0.013** ^*∗*^	**3.04** ^*∗∗∗*^
B	19	16.1	7	6.0			

^*∗*^Significant difference; ^*∗∗*^OR for genotypes AB/AA. CI = 0.991–7.104; ^*∗∗∗*^OR for alleles B/A. CI = 1.228–7.545.

**Table 2:** Risk estimate of bad genital hygiene behaviors in different MBL genotypes among RVVC cases and controls.

*MBL *genotype	Case *n* = (57)		Control *n* = (59)		Test of significance	*P* value	OR (95% CI)
	*n*	%	*n*	%			
AA							
Bad	28	66.7	19	36.5	**χ** ^2^ **8.436**	**0.004** ^*∗*^	**3.47** ^*∗∗*^
Good	14	33.3	33	63.5			

AB							
Bad	14	93.3	3	42.9	*Fisher's exact*	**0.021** ^*∗*^	**18.67** ^*∗∗∗*^
Good	1	6.7	4	57.1			

^*∗*^Significant difference; ^*∗∗*^OR for genital hygiene behaviors, bad/good. CI = 1.478–8.146; ^*∗∗∗*^OR for genital hygiene behaviors, bad/good. CI = 1.500–232.291.

**Table 3:** Comparison between wild type (AA) and mutant types (AB and BB) MBL serum levels among RVVC cases and controls.

Study group	MBL serum level (*µ*g/mL)			Test of significance	*P* value	
	AA	AB	BB			
RVVC cases (*n* = 59)	(*n* = 42)	(*n* = 15)	(*n* = 2)			
Mean ± SD	1.31 ± 0.88	0.69 ± 0.33	0.29 ± 0.28	*Kruskal-Wallis*	**0.011** ^*∗*^	**0.019** ^*∗∗*1^ **0.033**^*∗∗*2^ **0.265**^*∗∗*3^
Median (range)	1.02 (0.09–3.53)	0.67 (0.13–1.05)	0.4 (0.09–0.49)			

Controls (*n* = 59)	(*n* = 52)	(*n* = 7)				
Mean ± SD	1.35 ± 0.86	1.08 ± 0.96	-	*Mann–Whitney*	**0.23**	
Median (range)	1.15 (0.14–3.70)	0.51 (0.35–2.98)				

^*∗*^Significant difference; ^*∗∗*1^*P* value between AA and AB genotypes MBL concentrations; ^*∗∗*2^*P* value between AA and BB genotypes MBL concentrations; ^*∗∗*3^*P* value between AB and BB genotypes MBL concentrations.

## Data Availability

The data used to support the findings of this study are available from the corresponding author upon request.
